# Supplementary material for: Development of modified CTAB and Trizol protocols to isolate high molecular weight (HMW) RNA from polyphenol and polysaccharides rich pigeonpea (Cajanuscajan (L.) Millsp
Source: PLoS One. 2023 Dec 8;18(12):e0291949. doi: 10.1371/journal.pone.0291949 (PMC10707625; doi:10.1371/journal.pone.0291949)
Supplement: S1 Raw images — (PDF) [file pone.0291949.s001.pdf]

Figure: 1

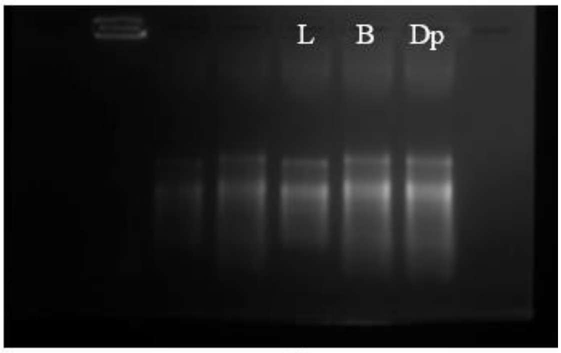

a

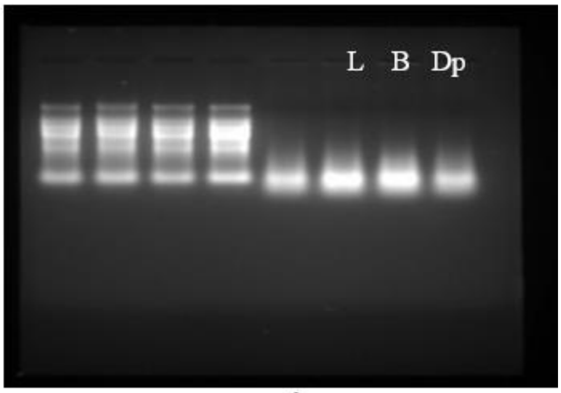

b

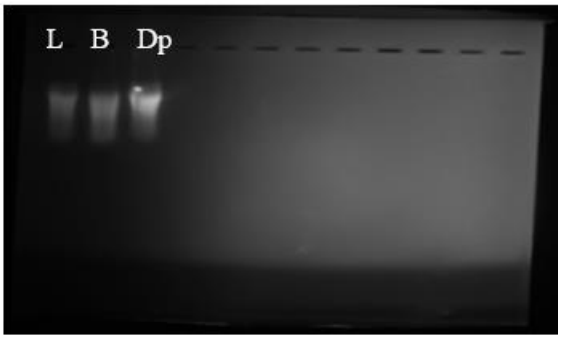

c

Fig.1 Denaturing formaldehyde agarose gel electrophoresis of RNA isolated from three different tissues (L- Leaf, B-Bud, Dp-Developing pod) of pigeonpea using different RNA isolation methods (unmodified). Fig. 1a- CTAB method, Fig. 1b- Trizol method, Fig. 1c- CTAB- LiCl method

Figure: 3

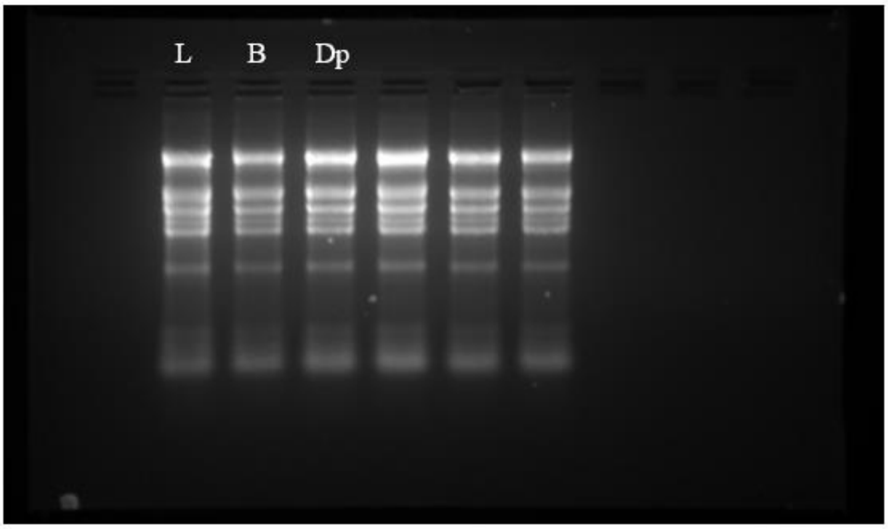

b

Fig.3 Denaturing formaldehyde agarose gel electrophoresis of RNA isolated from three different tissues (L-Leaf, B-Bud, Dp-Developing pod) of pigeonpea using modified CTAB (3a) and Trizol method (3b)

Figure: 4

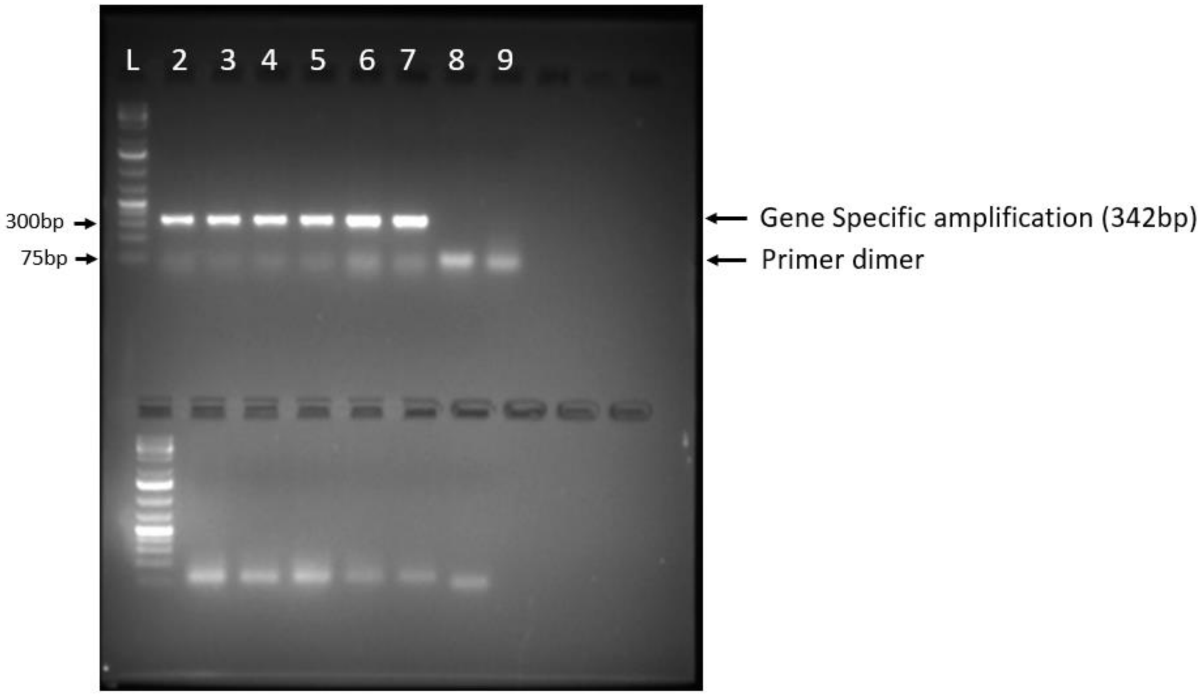

Fig.4 PCR amplification of Serpin protease inhibitor gene using cDNA prepared from RNA isolated by modified CTAB and Trizol method (Lane 1-1kb plus DNA Ladder, Lane 2-7 PI gene amplification, Lane 8- PCR amplification using RNA as the template, Lane 9 - control with water
