# Supplementary material for: Development of modified CTAB and Trizol protocols to isolate high molecular weight (HMW) RNA from polyphenol and polysaccharides rich pigeonpea (Cajanuscajan (L.) Millsp
Source: PLoS One. 2023 Dec 8;18(12):e0291949. doi: 10.1371/journal.pone.0291949 (PMC10707625; doi:10.1371/journal.pone.0291949)
Supplement: Attachment — Submitted filename: response to reviewers.docx [file pone.0291949.s002.docx]

To

**The Editor**

Plos One

Please find enclosed our detailed responses to the reviewer’s comments for the manuscript “Development of Modified CTAB and Trizol protocols to isolate High Molecular Weight (HMW) RNA from polyphenol and polysaccharides rich Pigeonpea (*Cajanus cajan* (L.) Millsp.”. We deeply acknowledge the Coordinating Editor for his/her nice coordination between the authors and reviewers.

The reviewer’s comments were very constructive and mainly concerned with (1) The steps followed to remove polyphenols and polysachharides during isolation of RNA (2) The applicability and novelty of the work (3) How the given protocols will work for RNA isolation from root sample and aquatic plants

We appreciate the time and effort that you and the reviewers dedicated to providing feedback on our manuscript. We have dealt with all of the critical comments in full and revised the manuscript.

We hope that you agree that we have satisfactorily dealt with the reviewer’s comments in full.

We are adding four names that have played significant roles during the work and have not added earlier while submission. Therefore, it is our humble submission to you to kindly accept our request and add these names too.

**Deepesh Kumar and Kuldip Jayaswall:** **Assisted to perform and optimize the steps.**

**Arvind Nath Singh and Sanjay Kumar: Helped during overall research work and correction of the manuscript.**

Dr. Deepanshu Jayaswal

Co-Corresponding author

**Response to editor:**

We did not receive any specific fund for this work.

We have all the dataset in the MS only. No specific dataset is required out of the MS.

The attached image of the gel electrophoresis is cropped only to remove other parts outside of the gel. The well and primer dimer can be clearly seen in the image. However, if raw figures will be required, we will upload that also.

**Response to reviewer 1 comments**

**Comment 1:** The article entitled "Development of Modified CTAB and Trizol protocols to isolate High Molecular Weight (HMW) RNA from polyphenol and polysaccharides rich Pigeonpea (Cajanus cajan (L.)" is well prepared. But the article doesn't say how the polyphenol and polysaccharides are removed from the sample. What are the chemicals/steps involved in the process of removal of polyphenol and polysaccharides.

**Response:** Thanks for reviewing the manuscript critically that will indeed improve the quality of the MS. As per as the chemicals/steps are concerned, We have used (1) PVP with the liquid nitrogen during crushing of the samples (2) The 2X volume of Trizol was used (containing 2 times guanidium thiocyanate) (3) Phenol Chloroform Isoamylalcohol was used rather than only Isoamylalcohol (4) the precipitation was done for 1 hour rather than 24 h (5) Washing step was repeated to remove excess salts

**Response to reviewer 2 comments**

**Comment 1:** Highlight the major outcome that differentiates the current protocol with the earlier reports.

**Response:** Thank you for the raising the outcome issues that will make the MS different to others. Following outcomes are in the MS.

(1) Yield sufficient and good quality of total RNA from all the tissues used in the study (leaf, flower and developing pod) ranged from 289 to 422μg per gram of tissue (Line 206, 207)

(2) Following the earlier protocols, RNA obtained were partially degraded and did not produce a reliable gene expression

(3) The RNA isolated by usual trizol method did not show any bands of 18S and 28S rRNA in the gel picture (Fig 1b), indicating that they were unable to precipitate high molecular weight RNA in the presence of high phenolic and carbohydrates compounds (Line 211 to 214).

In the concerned paragraph, all outcomes are mentioned. (Line number 206-224)

**Comment 2:** How efficient the current protocol with CTAB- LiCl method as it provides higher quality and quantity?

**Response:** The current method is more productive than existing CTAB-LiCL method, as it mentioned in terms of higher quality and quantity of RNA particularly in case of pigeonpea. However, the existing CTAB-LiCL method could also be useful for other crops.

Since a 1-hour isopropanol precipitation was utilised instead of an overnight LiCl precipitation, the proposed method required less time compared to CTAB-LiCl method while maintaining the same level of RNA quality and quantity. The reported protocols washing step was performed twice to remove excess salts, as opposed to the CTAB-LiCl method single washing step using 70% cold ethanol.

**Comment 3:** More literatures are available for CTAB-Trizol for Plant RNA isolation. What is the need of your work?

**Response:** I respectfully agree with the concerned raised. As per mentioned statements and figures in the manuscript, the existing methods were used to isolate RNA from Pigeonpea but somehow, the satisfactory results were not obtained. Therefore, the study was conducted to develop this modified method of RNA isolation.

**Comment 4:** Does this protocol is applicable to aquatic plants?

**Response:** We have not tried to isolate RNA from aquatic plants following this protocol as our research was on Pigeonpea but in our opinion, it could be used for aquatic plants.

**Comment 5:** What are the advantages and limitations of already available CTAB and Tizol method from your view with current work?

**Response:** Thanks. The advantage of already available CTAB and Tizol method will be as per the protocol tested on the concerned individuals. Here, in our case, the existing protocol was not too favorable, so this is the limitation of the existing protocol. Additionally, the modified steps and chemicals that we have used were limiting in the already available CTAB and Tizol method that made our study different from them.

The modified CTAB and trizol protocols yield sufficient and good quality of total RNA from all the tissues used in the study (leaf, flower and developing pod) ranged from 289 to 422 μg per gram of tissue. In contrast, unmodified protocols did not produce adequate amount of RNA, as the CTAB method produced RNA was partially degraded and 28S and 18S rRNA bands were not distinctly separated. The RNA isolated by usual trizol method did not show any bands of 18S and 28S rRNA in the gel picture, indicating that they were unable to precipitate high molecular weight RNA in the presence of high phenolic and carbohydrates compounds. The improved methods for the RNA isolation are rapid, efficient, easy and cost effective and can be used in the genetic studies in the molecular biology methods. Additionally, the modified protocols removed the excess of salt and co-purifying contaminants, which interfere in the subsequent applications and these protocols requires less time compared to conventional method.

**Comment 6:**  What are the other protocols that are available for terrestrial plants RNA isolation?

**Response:** SDS-phenol based, CTAB method, Trizol method and CTAB-LiCl methods are available for terrestrial plants RNA isolation.

**Comment 7:**  Why the author haven’t used RNA/DNA ladder in the FIgue 1&3?

**Response:** Figures 1 and 3 in the manuscript both showed RNA. The most important factors in determining the quality and amount of the material during RNA gel electrophoresis are the size of the bands (28S and 18S), the intensity of the bands, and the presence/absence of smears. Consequently, RNA/DNA ladder has not been taken into account for both figures.

**Comment 8:**   Did you tried to isolate RNA from root sample using this protocol?

**Response:** We are sorry to say that we have not tried to isolate RNA from root sample using this protocol but in our opinion it will work for sure as we are using PVP during crushing of the sample with liquid nitrogen so it will while using hard tissues like roots.
